# Congenital Hydrometrocolpos as a Diagnostic Dilemma: A Report of Two Cases From Benin City, Nigeria

**DOI:** 10.7759/cureus.91781

**Published:** 2025-09-07

**Authors:** Mary-Ann O Monyei, Edmund Orbih, David Osifo

**Affiliations:** 1 Pediatric Surgery, University of Benin Teaching Hospital, Benin City, NGA; 2 Pediatrics, University of Benin Teaching Hospital, Benin City, NGA

**Keywords:** congenital, diagnostic dilemma, hydrometrocolpos, nigeria, pediatric gynecology

## Abstract

Congenital hydrocolpos/hydrometrocolpos is a rare condition affecting the upper vagina/uterus and upper vagina, respectively, characterized by uterine and vagina dilation as a result of a distal obstruction to the flow of accumulated cervical mucus and secretions. The presentation of this condition may vary from abdominal pain with or without symptoms of urinary or gastrointestinal obstruction. These varied presentations may often confound and delay diagnosis. We present two cases of congenital hydrometrocolpos to highlight the diagnostic dilemma and increase the high index of suspicion.

Cases one and two involved a six-week-old infant and an eight-day-old neonate, respectively. Both were only correctly diagnosed intra-operatively with congenital hydrometrocolpos and were managed at this center within a two-year period. Both of them presented with huge cystic intra-abdominal masses and features of urinary and gastrointestinal tract obstruction. Imaging revealed a cystic intra-abdominal mass with a radiologic diagnosis of mesenteric cyst in both cases. They were both worked up and subsequently had an exploratory laparotomy, which revealed a markedly dilated uterus and upper vagina filled with turbid fluid and a transverse intra-luminal septum separating the vagina from the introitus. Intra-operative diagnosis of hydrometrocolpos was made in both cases. Transabdominal drainage of the hydrometrocolpos with utero-vaginostomy and two weeks of vagino-uterine stenting were successful in both cases. Accurate pre-operative diagnosis of this simple yet complex congenital anomaly is still a challenge in the sub-Saharan region. A high index of suspicion in all female neonates/infants presenting with intra-abdominal mass is advocated to improve early diagnosis and commencement of appropriate treatment.

## Introduction

Congenital hydrometrocolpos is a rare condition affecting the uterus and upper vagina. It is characterized by gross dilation of the uterus and vagina as a result of a distal obstruction to the flow and accumulation of cervical mucus [1]. Depending on the severity of the distension, it may manifest and be diagnosed in prenatal or neonatal life, or may remain undetected until early childhood or even puberty. This gross distention often results in varied presentations, most of which stem from obstruction to urinary flow or intestinal obstruction. There is no single "gold standard" test for the condition. Diagnosis often involves high clinical suspicion combined with imaging, such as MRI and ultrasound scan.

Historically, many reports of this condition were from stillbirths, with the diagnosis made at autopsy [2]. It is a relatively rare condition, with an incidence of 0.006% [1]. With the advent of higher-resolution prenatal ultrasound and access to more experienced sonographers in developed countries, a significant percentage of diagnoses are being made during the antenatal period, which has accelerated postnatal interventions and improved outcomes [2]. In many developing countries, affected children still present with severe complications due to late presentation in infancy or older. A literature search revealed that very few cases of congenital hydrometrocolpos have been documented in Nigeria and the West African sub-region. We present two cases that were managed within two years in our center to highlight the diagnostic dilemma and increase the high index of suspicion in female children presenting with an abdominal mass.

## Case presentation

Case 1

A six-week-old female infant presented at the children's emergency room with complaints of progressive abdominal swelling and recurrent vomiting since birth. The swelling was initially in the lower central abdomen and has rapidly increased upwards. At age three weeks, she began having non-projectile, non-bilious vomiting of recently ingested milk. These episodes gradually increased in frequency with resultant failure to thrive and dehydration. The mother had also noticed straining to urinate. She had an abdominal ultrasound scan done, which revealed an intra-abdominal cyst. Antenatal history was unremarkable, and ultrasound scans done at weeks nine, 15, and 36 gestation detected no abnormalities. She was delivered at 36 weeks via emergency cesarean section for severe pre-eclampsia with a birth weight of 3.2 kg. Examination on arrival at the emergency room revealed a moderately dehydrated, pale, and febrile (39°C) six-week-old infant, weighing 2.8 kg (had lost 400 g). The abdomen was markedly distended with a palpable mass that occupied almost the entire abdominal cavity and appeared to arise from the pelvis. She was anemic with a markedly elevated white cell count. Blood chemistry revealed metabolic acidosis and hypokalemia. Results are displayed in Table 1.

**Table 1:** Relevant CBC and serum electrolyte results at presentation to the emergency room. CBC: complete blood count

Variables	Laboratory result	Reference range
Hematocrit	19%	45-65%
Total white cell count (WBC)	21,000 cells/m^3^	4,000-12,000 cells/m^3^
Serum bicarbonate (HCO_3_)	12 mmol/L	20-30 mmol/L
Serum potassium	2.7 mmol/L	3.5-5.5 mmol/L

She was fluid resuscitated, given calculated doses of potassium chloride and sodium bicarbonate, started on intra-venous antibiotics, and worked up for surgery following a repeat abdominopelvic ultrasound scan on arrival, which diagnosed an extensive thick-walled complex cyst (312 mL), which was concluded to be a mesenteric cyst. This delayed surgery up to two weeks. Pre-operative echocardiogram revealed no structural abnormality.

Intra-operative findings included a distended uterine cavity and upper vagina cavities filled with 450 mL of seropurulent fluid, a blind-ended vagina lumen with a thick-walled transverse septum separating it from the introitus. Uterine and upper vaginal cavities were drained trans-abdominally, and a utero-vaginostomy was done by excising the septum and inserting a size 18Fr silicone catheter to serve as a stent and for continuous drainage for two weeks. Apart from the surgical site infection, which responded to treatment, the post-operative period was uneventful, and she was subsequently discharged for post-operative follow-up in the surgical outpatient clinic.

Case 2

An eight-day-old female neonate was brought in from a referral facility with multiple abdominal masses noted in a prenatal scan just prior to delivery at 37 weeks, and an inability to pass urine since birth. Antenatal history was otherwise unremarkable. The mother had a scan at 12 weeks, which did not show the abnormalities noted later.

**Figure 1:**
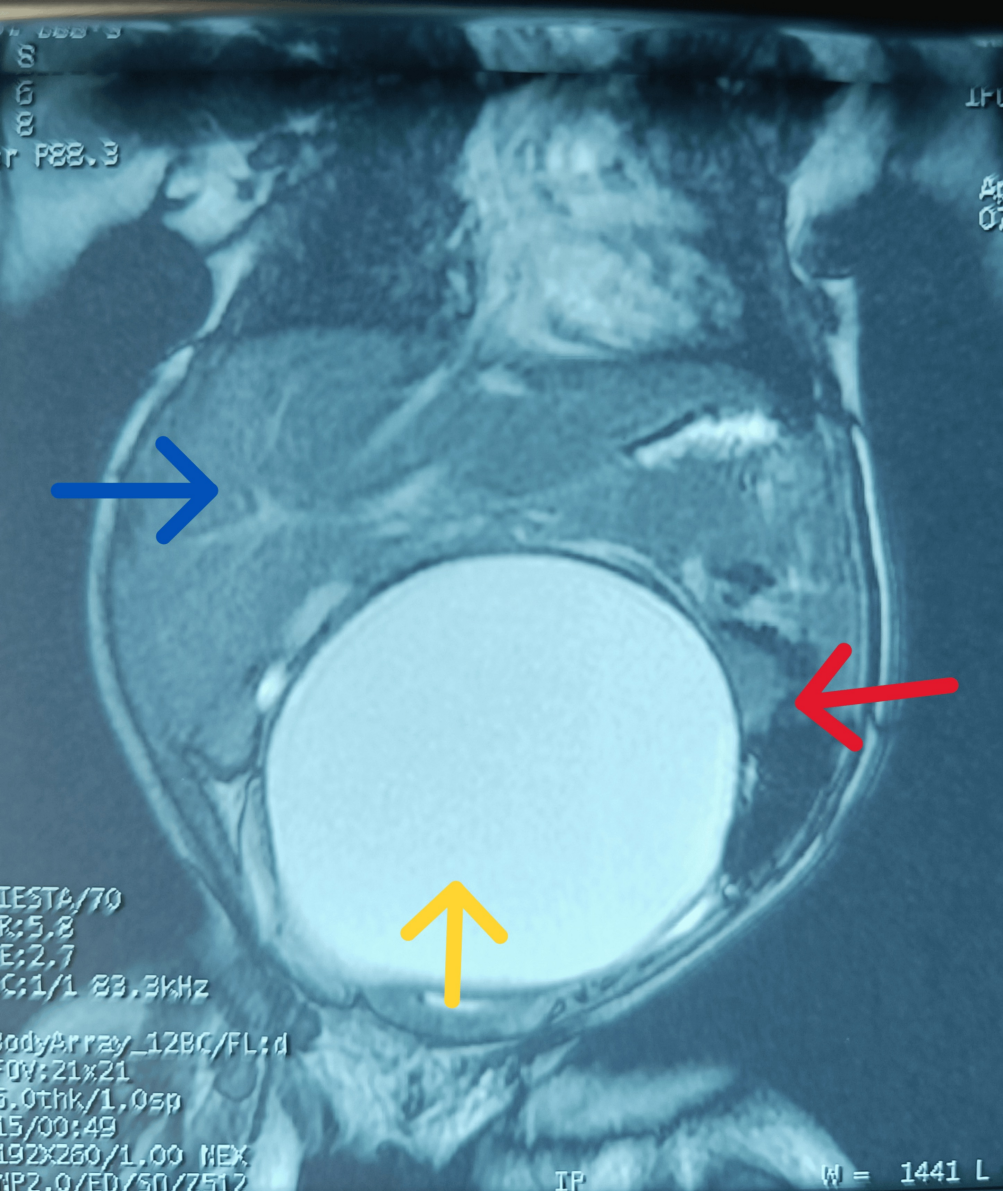
T2-weighted magnetic resonance imaging of the thoracoabdominal region (coronal view). The image shows an 8.1x3.5 cm large well-defined fluid-filled sac (yellow arrow), displacing the bowel loops (red arrow) and liver (blue arrow). A pre-operative diagnosis of an omental cyst was made.

She was otherwise well-looking at presentation with urethral catheter in situ, draining clear urine. A palpable intra-abdominal mass extended from the pelvis to midway between the xiphisternum and umbilicus. It was immobile on the vertical axis but could be moved from side to side. There was a normally sited and patent anal orifice with no visible introitus and perineal bulge. A T2-weighted MRI image showed an 8.1x3.5 cm fluid-filled, large, well-defined sac, displacing bowel loops and liver, suspected to be a mesenteric/omental cyst (Figure 1).

**Figure 2:**
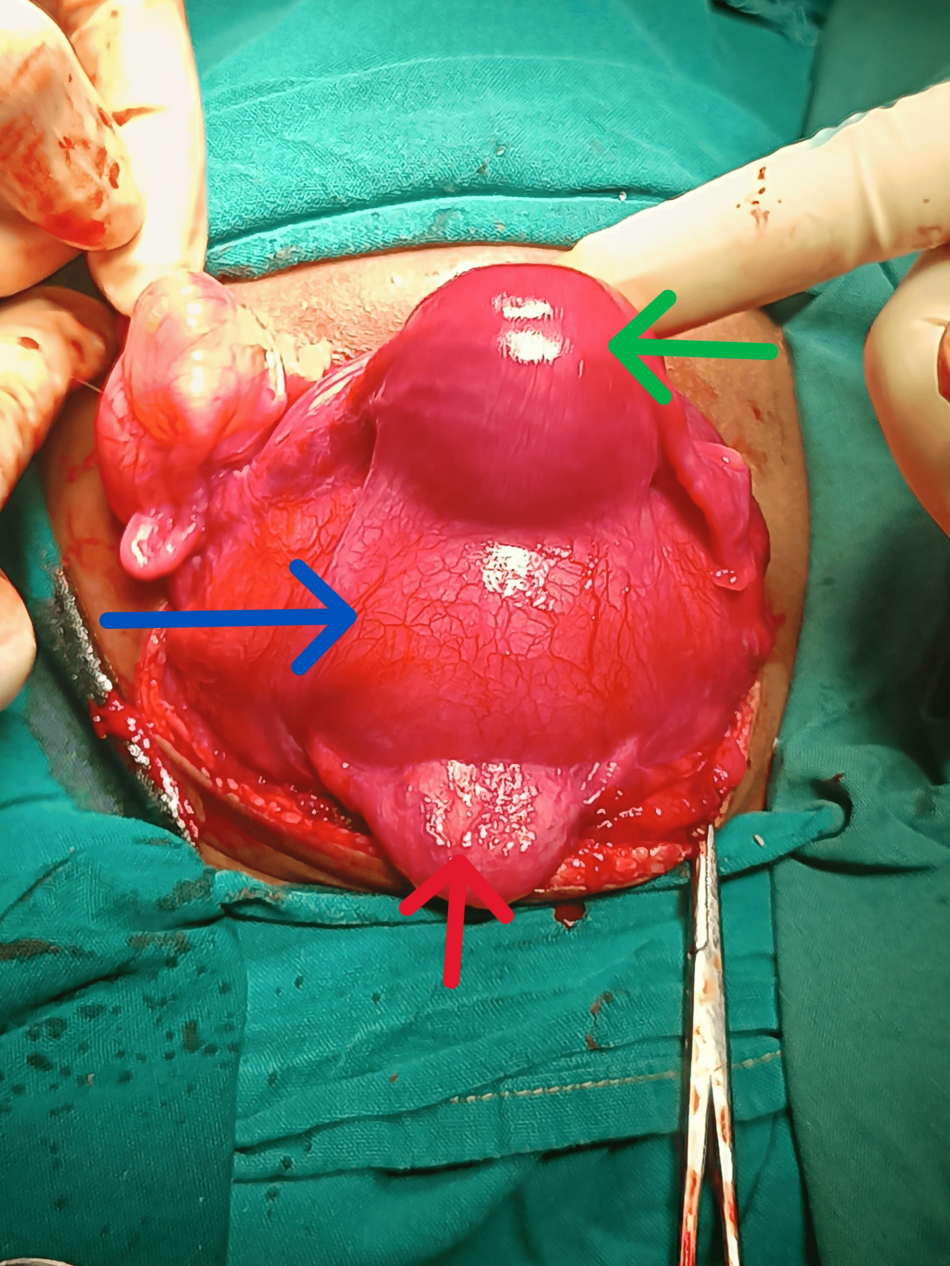
Intra-operative photograph of the hydrometrocolpos. The urinary bladder (red arrow), with ureters inserting, lies anterior to the distended vagina (blue arrow) and uterus (green arrow).

She subsequently had an exploratory laparotomy at two weeks of life, with findings of a markedly dilated uterus and vagina containing 200 mL of fluid, abutting the urinary bladder and obstructing the urethra anteriorly, with a blind-ending upper vaginal pouch (Figure 2).

Transabdominal drainage of hydrometrocolpos and utero-vaginostomy was done by excising the septum and inserting a size 18Fr silicone catheter to serve as a stent and continuous drainage for two weeks post-operatively. The post-operative course and follow-up in the surgical outpatient clinic have been uneventful.

## Discussion

The two cases of hydrometrocolpos managed in this center within two years presented a diagnostic dilemma, with accurate diagnosis made intra-operatively, similar to what is reported by other studies [3]. Despite the diagnostic challenge, the outcomes of surgical intervention were rewarding with uneventful post-operative follow-up in an outpatient clinic.

Congenital hydrometrocolpos is a rare disorder (1:16,000 female newborns) [4]. Only a few cases have been documented in Nigeria [3]. It is believed that the fluid which accumulates in the uterus and vagina arises from secretions by the cervical mucus glands following stimulation by maternal hormones during pregnancy [5]. The condition may be associated with a distal obstruction to the flow of these accumulated secretions, seen in the two index cases. Labial adhesion, persistent urogenital sinus/cloacal anomaly, vaginal agenesis (McKusick-Kaufman syndrome), transverse vaginal septum (as seen in the index cases), and imperforate hymen have been reported as possible causes of vaginal obstruction leading to hydrometrocolpos [6,7]. Occasionally, a persistent cloaca, urogenital sinus, or an extrinsic compressive mass like a sacrococcygeal teratoma, which may or may not be apparent from the exterior, may be the cause of vaginal obstruction [8].

With improvements in prenatal ultrasound scans, many of these patients are being diagnosed before birth with the immediate post-natal institution of appropriate treatment, resulting in the best outcomes. In sub-Saharan Africa, however, most of these cases are still undiagnosed both at the prenatal and neonatal periods, resulting in late infancy or intra-operative diagnosis as seen in the first index case. Others in similar settings have reported occasional mortality due to delay in the diagnosis with resultant complications [3,9]. This may be attributed to the paucity of experienced sonographers and the rarity of the condition in many settings. The mode of presentation is dependent on the degree of compression of the surrounding structures and the duration of symptoms. This large accumulation may lead to urologic and gastrointestinal complications, the most common of which is bladder compression leading to hydronephrosis and renal compromise, as seen in the second index case. The hydrometrocolpos may cause symptoms of partial intestinal obstruction as seen in the first index case and reported by others [3,9]. Diagnostic delay, particularly in older children, may also lead to physiologic derangement and poor general outcomes [9]. Therefore, it is important to suspect hydrometrocolpos in all female neonates with an abdominal mass, irrespective of other symptoms. Additionally, a thorough newborn examination will help in the early detection of abnormalities that cause vaginal obstruction. Ultrasound scans and magnetic resonance imaging currently constitute the investigations of choice [10].

Treatment is surgical and depends on the cause as follows: hymenectomy in imperforate hymen, vaginal pull-through in vaginal atresia, application of estrogen cream in labial adhesions, excision of transverse vaginal septum; as done in index cases, as well as excision of sacrococcygeal teratoma are surgical options. Transabdominal drainage of the hydrometrocolpos is preferred to the transvaginal route to prevent reaccumulation [5].

## Conclusions

Congenital hydrometrocolpos, although rare, presented a diagnostic dilemma as the reported index cases were only accurately diagnosed intra-operatively. Although outcomes of surgery are rewarding, early diagnosis requires a high index of suspicion, which should be aided with accurate ultrasonography and magnetic resonance imaging, which were limited during the period. It is advocated that all newborn females with abdominal mass, with or without symptoms of gastrointestinal or urinary tract obstruction, should be suspected and thoroughly evaluated for hydrometrocolpos. This will result in the prompt institution of appropriate surgical intervention to reduce complications of neglected hydrometrocolpos and prolonged urinary and gastrointestinal obstruction sequelae.
